# Supplementary material for: Splice-Junction-Based Mapping of Alternative Isoforms in the Human Proteome
Source: Cell Rep. Author manuscript; Available in PMC 2020 Jan 15. (PMC6961840; doi:10.1016/j.celrep.2019.11.026)
Supplement: 2 [file NIHMS1546469-supplement-2.zip › DF1/PXD010154/Esophagus/Esophagus_5_PALLD_NIQEPEEETANQIYWFK.pdf]

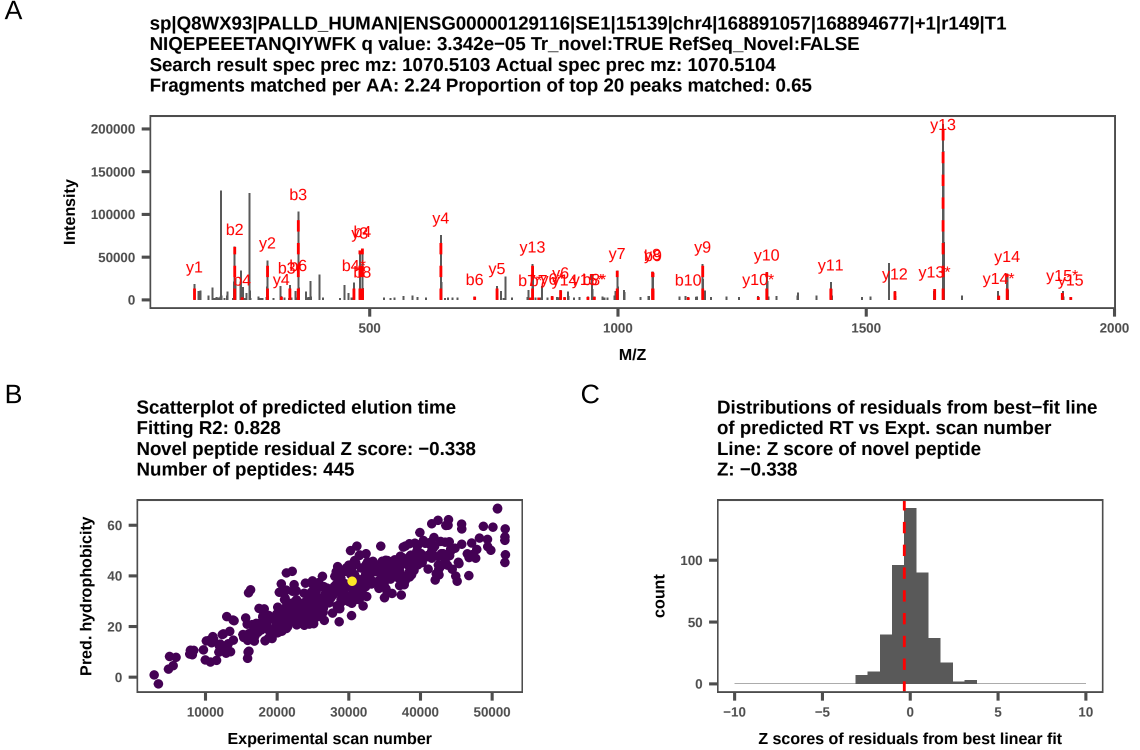
A
sp|Q8WX93|PALLD_HUMAN|ENSG00000129116|SE1|15139|chr4|168891057|168894677|+1|r149|T1
NIQEPEEETANQIYWFK q value: 3.342e−05 Tr_novel:TRUE RefSeq_Novel:FALSE
Search result spec prec mz: 1070.5103 Actual spec prec mz: 1070.5104
Fragments matched per AA: 2.24 Proportion of top 20 peaks matched: 0.65
Intensity
200000
150000
100000
50000
0
500
1000
1500
2000
M/Z
y1
b2
b4
y2
b3
y4
b6
y4
b6
y5
y13
y7
y9
y10
b10
y10*
y11
y12
y13
y13*
y14
y14*
y15*
y15
B
Scatterplot of predicted elution time
Fitting R2: 0.828
Novel peptide residual Z score: −0.338
Number of peptides: 445
Pred. hydrophobicity
60
40
20
0
10000
20000
30000
40000
50000
Experimental scan number
C
Distributions of residuals from best−fit line
of predicted RT vs Expt. scan number
Line: Z score of novel peptide
Z: −0.338
count
100
50
0
−10
−5
0
5
10
Z scores of residuals from best linear fit
